# *Stylus*: A System for Evolutionary Experimentation Based on a Protein/Proteome Model with Non-Arbitrary Functional Constraints

**DOI:** 10.1371/journal.pone.0002246

**Published:** 2008-06-04

**Authors:** Douglas D. Axe, Brendan W. Dixon, Philip Lu

**Affiliations:** Biologic Institute, Redmond, Washington, United States of America; University of Cape Town, South Africa

## Abstract

The study of protein evolution is complicated by the vast size of protein sequence space, the huge number of possible protein folds, and the extraordinary complexity of the causal relationships between protein sequence, structure, and function. Much simpler model constructs may therefore provide an attractive complement to experimental studies in this area. Lattice models, which have long been useful in studies of protein folding, have found increasing use here. However, while these models incorporate actual sequences and structures (albeit non-biological ones), they incorporate no actual functions—relying instead on largely arbitrary structural criteria as a proxy for function. In view of the central importance of function to evolution, and the impossibility of incorporating real functional constraints without real function, it is important that protein-like models be developed around real structure–function relationships. Here we describe such a model and introduce open-source software that implements it. The model is based on the structure–function relationship in written language, where structures are two-dimensional ink paths and functions are the meanings that result when these paths form legible characters. To capture something like the hierarchical complexity of protein structure, we use the traditional characters of Chinese origin. Twenty coplanar vectors, encoded by base triplets, act like amino acids in building the character forms. This vector-world model captures many aspects of real proteins, including life-size sequences, a life-size structural repertoire, a realistic genetic code, secondary, tertiary, and quaternary structure, structural domains and motifs, operon-like genetic structures, and layered functional complexity up to a level resembling bacterial genomes and proteomes. *Stylus* is a full-featured implementation of the vector world for Unix systems. To demonstrate the utility of *Stylus*, we generated a sample set of homologous vector proteins by evolving successive lines from a single starting gene. These homologues show sequence and structure divergence resembling those of natural homologues in many respects, suggesting that the system may be sufficiently life-like for informative comparison to biology.

## Introduction

Because of their simplicity, lattice polymer models (where structures consist of chains of connected beads occupying neighboring positions on a two- or three-dimensional lattice) have become attractive artificial systems for studying certain general properties of structure-forming polymers. The study of protein evolution, particularly the origin of protein folds, is one challenging area where lattice models have been employed [1]–[3]. Although these model constructs are unrealistic in many respects, they do provide computationally tractable sequence spaces that can be mapped onto structure spaces with specified mapping rules. As such, they form a class of systems that can be studied in their own right, providing insights that (with due care) will continue to advance our understanding of real biological problems [1].

One such insight is that protein-like models (in contrast to RNA models) tend to show sparse connectivity between regions of sequence space that encode different structures [1]. In other words, stepwise paths through sequence space that accomplish a structural transformation without passing through unstructured intermediates appear to be rare. This clearly fits expectations for real proteins, where reorganization of core structure would seem to require complete loss of structure (and therefore function) along the way [4]. It also fits experimental observations, which show that the expected deterioration is common not only for transitions between different folds [5] but also, more surprisingly, for transitions between different sequences encoding the same fold [6].

What it fails to fit well, at first glance anyway, is the pattern of structural similarities evident in natural proteins. If there is a substantial probabilistic barrier to structural innovation in the protein world, then we might expect the evolutionary process to make do without it. By this view, the protein world ought to consist of one structural archetype put to many different uses, each involving modest alteration of peripheral structure but no major reorganization of the fold. Subsets of the natural proteins show precisely this, but the whole picture is strikingly different. Here we find a surprising preponderance of “orphan” folds—folds that each occupy their own patch of structure space, well removed from everything else [7]. Although models have so far failed to explain how orphan folds can be so common [3], they have offered explanations for substantial structural radiation.

However, the models purporting to explain structural radiation generally use simplistic representations of selectable function. As Zeldovich *et al*. point out, many evolutionary models lack any causal connection at all between sequence and function [8]. But even when causal models are used, they tend to be simplistic. Hirst has discussed the various aspects of structural soundness (e.g., folding stability or speed) that are singled out as proxies for selectable function [9]. Recognizing the distinction between structural soundness and functional utility, he required lattice structures to form a pocket (analogous to an active-site cleft) in order to be deemed functional [9]. This was certainly a step in the right direction, but the underlying problem remains: While these properties are all necessary for the function of real proteins, they are not sufficient. If they were, one good structure would suffice, whereas in reality we see not only a great variety of structures but also a strong connection between this variety and the great variety of specific functions they perform.

Oversimplification of function tends to obscure this fundamental connection. As an example, consider the recent lattice study of Zeldovich *et al.*, which ties a genome's fitness to the lowest stability of its encoded proteins [8]. Their model enables a population carrying the gene for a single lattice structure to diversify to the point where evolved structures span the entire space of possibilities. But it achieves this not only by using stability as a proxy for function, but also by dispensing with the notion of a stability threshold—a minimal stability, below which structures are deemed non-functional [8]. In the end, structure space is freely explored here because it is entropically favorable for it to be explored, making structural variety an entropic artifact rather than a functional necessity. Because one good structure really does suffice in such a world, it seems unlike the real world, where “the great functional capacity and importance of proteins largely stems from the remarkable ability of these polymers to adopt distinct 3-dimensional structures” [3].

Can a new model be framed so as to capture this fundamental aspect of biology? A key step in this direction may be to base it on real function rather than a definitional substitute for function. Because real functions involve both specificity and real constraints, this would guarantee a level of functional realism that is not otherwise easily achieved. This principle is demonstrated by artificial-life simulations, like Avida [10], where computational tasks must be performed in order to gain a selective advantage. But because these tasks are performed by instructions rather than structures, Avida does not readily lend itself to protein studies.

Despite their limitations, though, all of the models discussed have strengths to offer. Furthermore, these strengths suggest a way to overcome the primary limitations. In particular, a model that ties real functions to polymer-like structures would have the potential to achieve a new level of biological realism. By incorporating real, specific functions it would be grounded in real functional constraints, and by basing these functions on polymer-like structures it would have a clear connection to real proteins. Here we describe such a model and introduce an open-source computational system that implements it, providing a complete environment for evolutionary experimentation on model genes that resemble bacterial genes.

## Results

### Model

#### Core Analogy

Human language shares several interesting properties with biology. Both use complex structures to perform complex functions, the complexity in both cases being hierarchical—high-level functions and structures being built from those on a succession of lower levels. And while they clearly operate within functional constraints, neither has the highly rigid structure of formal systems like computing languages. Rather, they are characterized by an abundance of rules, virtually none of which is absolute. Add to this the fact that both biology and language have been shaped by real populations with real complex histories, and the similarities are seen to be quite extensive.

A more specific analogy between proteins and written language has long been recognized [11]–[13]. A common approach here is to compare alphabetic strings to amino-acid chains, the first having the capacity for linguistic meaning and the second for biological function. But despite the obvious appeal of this comparison, important dissimilarities exist. Perhaps the most striking of these is seen in the very different effects of cumulative sequence change. When protein-coding genes are subjected to occasional mutations over long time periods, they manage to undergo substantial sequence change while maintaining their original function. Alphabetic sequences, on the other hand, are rapidly degraded by typographic substitutions, leading eventually to complete loss of function.

This dissimilarity appears to stem from a difference in the underlying causal relationships. In the protein world, functions are a direct consequence of physical structures. This, in combination with a highly many-to-one mapping of protein sequences to structures, allows sequences to change continually while meeting the structural constraints imposed by the original function (a phenomenon known as neutral drift [14]). In contrast, alphabetic strings function as raw sequences, with no physical structure mediating between them and their function (they are, of course, recorded and conveyed through physical media, but the only requirement for achieving this is accurate representation of sequence). Although alphabetic sequences show a many-to-one mapping to function, it is “many” in a sparse and highly discontinuous sense. Because these sequences are directly constrained by the rules of linguistic function (grammar, vocabulary and spelling) they cannot withstand the continual step-wise change seen in proteins.

**Figure 1:**
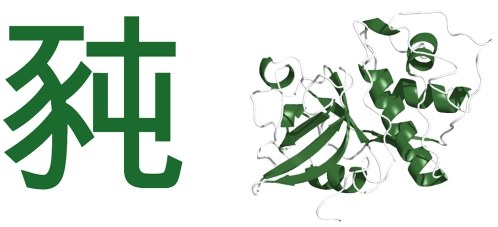
Structural analogy between Han characters and protein folds. This two-part character (identified by its hexadecimal Unicode number, U+8C58) is reminiscent of two-part protein folds like the one shown (PDB 1CQD).

The importance of structure in the protein world suggests that a structure-based system of writing would provide a better analogy. Many of the Asian languages use non-alphabetic writing based on the Chinese characters that became standardized during the Han Dynasty (206 BC–220 AD). Like alphabetic letters, the Han characters are recognized by their distinct structural forms. But unlike letters, the characters have word-like meanings as stand alone entities. In these written languages, then, basic linguistic meaning is rooted in structure rather than sequence. This suggests a new way of framing the linguistic analogy to proteins. Instead of viewing the letters in alphabetic strings as being analogous to the amino-acid residues in a protein chain, the new approach views the Han characters as being analogous to whole protein folds (Figure 1).

**Figure 2:**
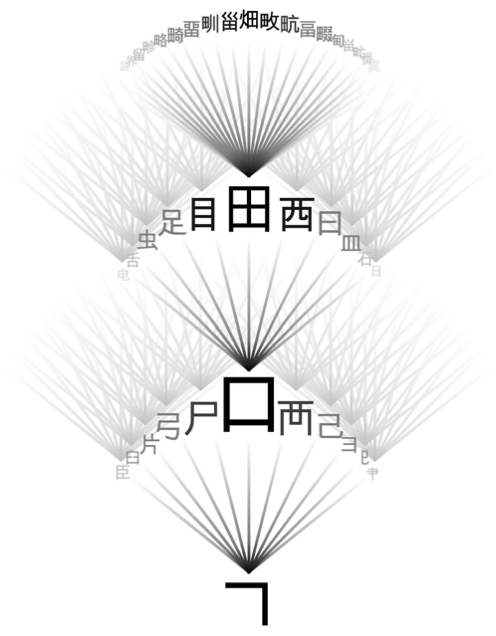
Hierarchical structure of Han characters. Single strokes, like that shown at the bottom, are combined to form successively more complex structures (shown as ascending layers). Characters range in complexity from a single stroke to dozens of strokes.

This structural connection carries a number of other similarities with it. Some of these will be mentioned briefly here, with more detailed discussion to follow. First, while the previous analogy provided approximate correspondence between the number of alphabetic letters and the number of amino acids, the new one provides approximate correspondence between the number of Han characters and the number of distinct protein folds or functions in the biosphere. The standard enzyme classification scheme, for example, covers just over four thousand known enzyme functions (http://www.enzyme-database.org/stats.php) which depend upon a few thousand family-level structures (http://scop.mrc-lmb.cam.ac.uk/scop/count.html#scop-1.71). By way of comparison, the Unihan database (http://www.unicode.org/charts/unihan.html) indicates that roughly five thousand Han characters find use in a single language (based on the number of characters with kFrequency tags, indicating use in traditional Chinese USENET postings; http://www.unicode.org/Public/UNIDATA/Unihan.html). Visual discrimination of so many characters requires structural complexity beyond that of alphabetic characters, approaching the complexity of protein folds in some respects. Figure 1 illustrates the rough similarity in the number of parts (the line or curve segments that form strokes compared to the elements of secondary structure) that compose whole characters and whole proteins. Finally, both worlds exhibit hierarchical structure, meaning that complex forms are built from successively simpler forms (see Figure 2), most of which find extensive reuse in a variety of combinations for a variety of functional ends.

#### Building on the Analogy

The new model is based on the real relationship between structure and function exhibited by the Han characters. These characters are not intrinsically polymer-like, but since they are written by moving a pen tip along a path, the extension to a polymer chain model is straightforward.

**Figure 3:**
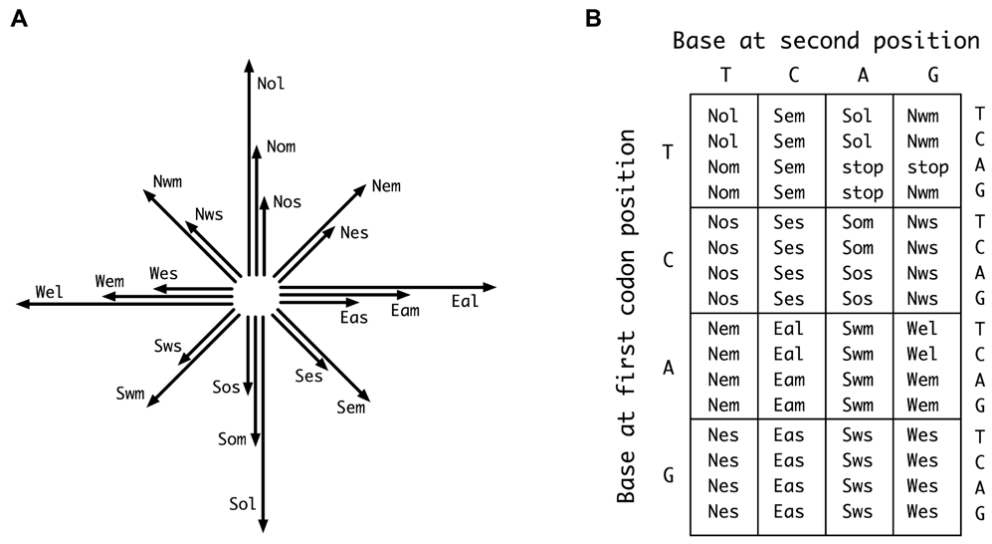
Monomers and genetic code for construction of model proteins. A) The set of vector monomers, named according to compass direction and length (i.e., *Nem* indicating a northeast vector of medium length). To ensure that vector addition produces different results with different vector combinations, small vectors are of length 1, medium vectors of length *e* ^1/2^ (≈1.65), and long vectors of length *e* (≈2.72). B) A standard genetic code for specifying the monomers with nucleotide triplets. Like the natural code [15] this code incorporates several features that reduce the impact of point mutations. These include extensive use of third-position degeneracy, strong correlation of second position with a key physical property (direction), and underrepresentation of vectors that would be most disruptive as substitutes (long vectors).

**Genetic Code.** Although the process of writing involves three-dimensional paths, written forms are more like two-dimensional paths (part inked and part invisible). Because of this, we have restricted our model to two dimensions. The geometric simplicity of two-dimensional paths calls for a very simple suite of monomeric building blocks. For this purpose we use twenty coplanar vectors of three possible lengths, aligning with the eight compass directions (Figure 3A). The numerical equivalence to the set of protein-forming amino acids allows a genetic code to be defined for the vector world, whereby vector sequences are encoded by base triplets in much the same way that genes encode amino-acid sequences (Figure 3B).

**Genes.** The artificial genes used in the vector world look just like textual representations of bacterial genes: they begin with an ATG start codon, proceed through any number of vector-encoding codons (the same 61 sense codons used in biology), and terminate with one of the three biological termination codons (TAA, TAG, or TGA).

**Figure 4:**
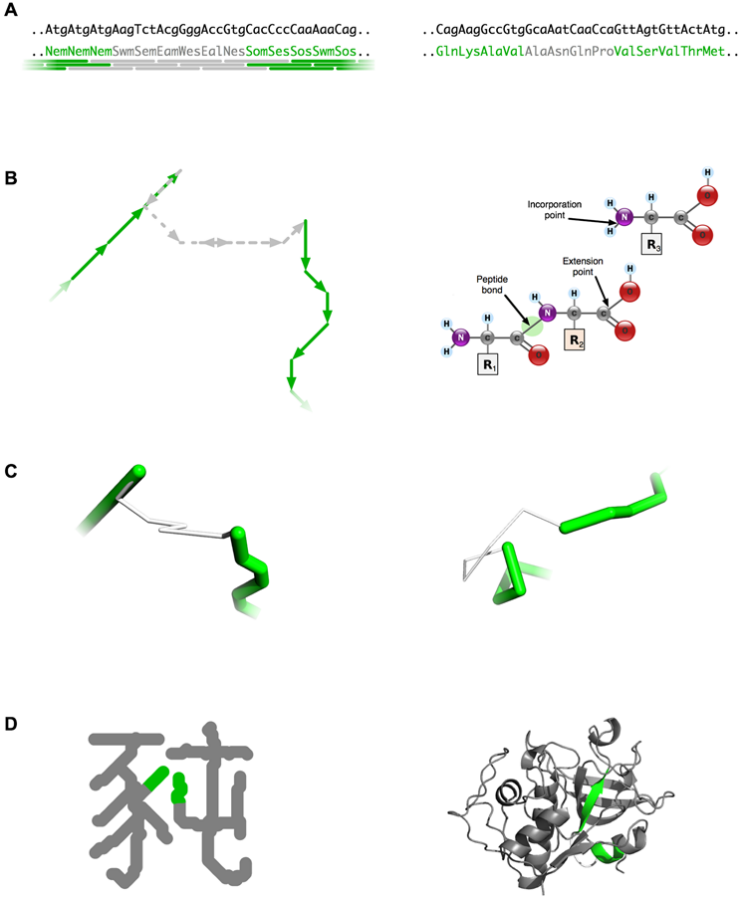
Parallels between vector-world and real-world protein synthesis. Steps are illustrated for a vector protein (U+8C58) on the left, with analogous aspects of a real protein (PDB 1CQD) on the right. A) Codons in an open reading frame specify monomers (vectors or amino acids) that may form regular local structure (green) or irregular local structure (grey). In the vector world a simple rule determines which is the case: A vector becomes part of regular structure if and only if it forms a coherent vector triplet (indicated by green tiles below the sequence; see text). B) Vectors are joined to form paths with head and tail termini, just as amino acids are joined to form chains with amino and carboxyl termini (right panel derived from public domain images by Yassine Mrabet). C) Vector proteins consist of strokes (formed by runs of coherent vectors) joined by moves (formed by runs of incoherent vectors), in much the same way that real proteins consist of units of secondary structure joined by turns or loops. D) Final working forms, highlighting the segments shown above.

**Primary structure: Vector sequences analogous to amino-acid sequences.** Just as real protein chains are built by addition of amino acids at the C-terminus, so vector proteins are built by joining the tail of the newest vector to the head of the previous one. In both worlds the gene product is a chain of linked monomers, each internal monomer having one point where it was added to the growing chain and another where the next addition was made (Figure 4A,B).

**Secondary Structure: Coherent path segments analogous to regular structure.** Folded protein chains consist of segments with regular backbone structure (primarily α helix or β strand conformations) connected either by turns or by segments with irregular structure (loops). For a vector protein to form a written character, it must likewise consist of segments of two types: those forming strokes, and those forming moves between strokes. The rule used to differentiate these resembles the distinction between regular and irregular backbone structure in proteins, in that both depend only on local chain conformation. In the protein world, secondary structure is indicated by a succession of residues with dihedral angles characteristic of either α helices or β strands. As shown in Figure 4, whenever three consecutive vectors in a vector protein have directions spanning an angle of 90 degrees or less (meaning the compass directions lie within a quarter of the circle) they are shown as visible line segments in the working form of the protein (i.e., the ready-to-read representation, as in Figure 4D–left). Portions of a vector protein that do not meet this condition are not shown in the working form, thereby allowing drawn strokes to be joined by undrawn moves between strokes. Because the 90-degree condition amounts to a test of local directional coherence, the terms *coherent* and *incoherent* are applied to vectors that pass or fail this condition, respectively.

**Figure 5:**
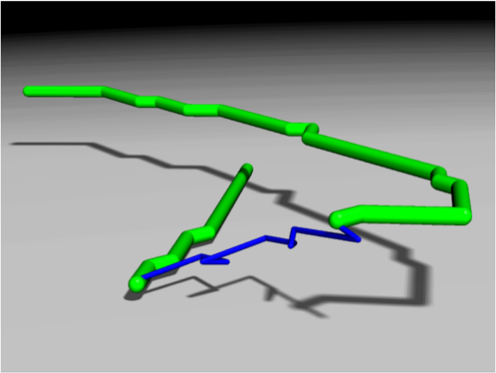
Layered 2D representation of vector proteins. Strokes (green) are placed on successively higher planes by rendering moves (blue) with a vertical component added to each vector.

**Dimensionality: Layered 2D analogous to 3D.** Real protein structures are three dimensional, whereas the vectors used to build vector proteins lie within a single plane. Still, clear visualization of the constituent vectors in a vector protein typically calls for enhanced representation in three dimensions. A useful way to produce pseudo-3D representations is to preserve the planar character of each stroke while expanding moves by adding a constant lift to every incoherent vector. As shown in Figure 5, this effectively maximizes visibility by stacking the strokes on layered planes spanned by moves. This approach will be used to visualize complete vector chains with the understanding that the working form (2D with incoherent vectors invisible) provides the connection to function.

**Figure 6:**
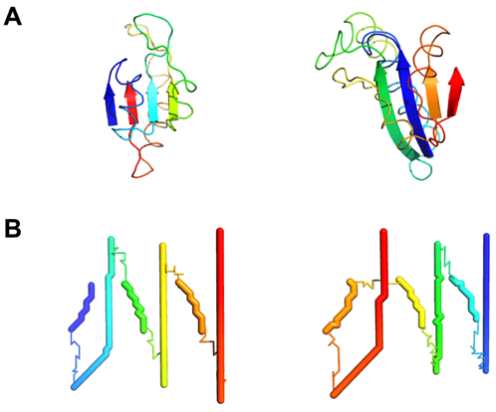
Chain topology in real and vector proteins. A) Sheet regions of 1VHR (left) and 1D1Q (right) with color running from blue to red in the amino-to-carboxyl direction. B) Vector proteins that perform the function of 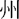 (U+5DDE) by means of different topologies, colored blue to red in the tail-to-head direction.

**Tertiary structure: Vector paths analogous to backbone structures.** Protein tertiary structure is characterized not only by the spatial arrangement of secondary structure elements but also by topology—how these elements are connected. Figure 6A illustrates this with two four-strand β sheets. Although the two sheets differ in geometric details like strand length and curvature, the color patterns highlight a more fundamental topological difference: the strands are ordered differently along the protein chains. Another key aspect of tertiary structure pertaining to sheets is strand direction, which may be parallel (i.e., uniform, as in this example), antiparallel, or mixed. All of these topological aspects of tertiary structure—arrangement, direction, and connectivity—have direct parallels in the vector world. For example, Figure 6B shows two vector proteins that both arrange their strokes in the form of 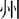 (U+5DDE), but they do so by means of different stroke directions and orders. Like the alternative sheet structures of Figure 6A, these vector proteins have fundamentally different tertiary structures.

How many distinct tertiary structures are possible? No clear way of answering this exists for real proteins, though it has been suggested that far more are possible than have been put to use in the biosphere [16]. Of all possible structures, some fraction would be capable of performing the biological functions of the natural proteins. Even if this fraction is small, it may include a great many more folds than the natural ones.

The relative simplicity of the vector world enables some of these numbers to be calculated. The number of fundamentally different ways for a vector protein to perform the function of 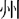, for example, is 46,080 (the number of ways to order the 6 strokes, multiplied by the number of ways to vary the direction—up or down—through a specified stroke order). For a character with *n* strokes, the number of alternatives is 2*^n^*·*n*!, which grows very rapidly as *n* increases: 3840 alternatives for 5 strokes, a million-fold more for 10 strokes, and ten-million-fold more again for 15. Considering that 9 or 10 strokes is a rough average for the set of characters in common use (9-stroke median, 10-stroke mean, based on the set of characters assigned USENET frequencies of 1, 2, or 3 in the Unihan database; http://www.unicode.org/Public/UNIDATA/Unihan.html), it is clear that the number of distinct vector folds that perform the function of any Han character vastly exceeds the number of characters.

**Figure 7:**
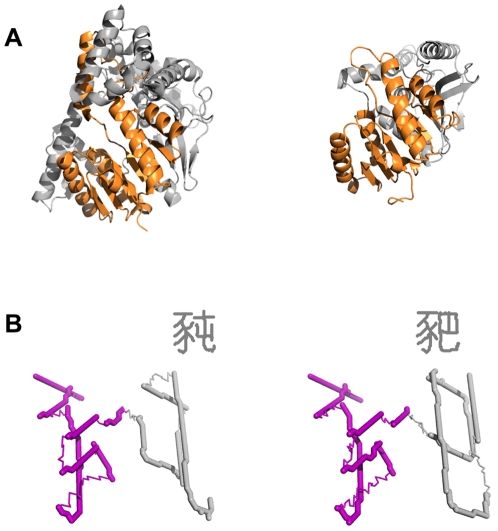
Domains as sub-structures with sub-functions. A) Two proteins that use similar NAD-binding domains (orange). Left: *α*-glucosidase monomer from *Thermotoga maritima* (PDB 1OBB). Right: L-lactate dehydrogenase monomer from *Bacillus stearothermophilus* (PDB 1LDN). B) Two vector proteins that use similar 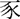 domains (purple) as described in text.

**Fold organization: Vector-protein domains analogous to protein domains.** Real proteins with more than about 150 amino-acid residues tend to fold with secondary structure grouped into two or more regions. In some cases it appears that these regions correspond to folding domains—portions of the protein chain that fold as independent units [17]. Sequence and structure comparisons across diverse protein families likewise suggest that proteins are composed of multiple parts. A domain-sized part of one protein is often found to have counterparts in other structural contexts, suggesting that structural and functional modularity have enabled evolutionary recombination of parts [18], [19]. An example of this is the NAD-binding domain, shown in two of its structural contexts in Figure 7A.

Because the Han characters have their own evolutionary history, with structural and functional modularity playing a major role, the vector world described here inherits these features. To retain these historical characteristics, the vector world is based on the traditional character forms used in Hong Kong and Taiwan (simplified versions of many of these characters being used in China and elsewhere). Figure 7B shows two vector proteins (functioning as 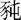 [U+8C58] and 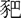 [U+8C5D]) that share a group of strokes. Like numerous other groups, this one performs a sub-function that appears in many structural combinations, making it akin to a protein domain. In this case the sub-function is that of 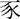 (U+8C55), which means *pig*. As is often the case for proteins, the composite functions show similarity that derives from the shared structural component: 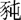 means *small pig*, and 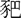 means *sow*.

Both worlds show considerable variation in how domains fit together to form multi-domain structures. For example, one of the NAD-binding domains (Figure 7A, left) is considerably more entangled with its complementing domain than the other, implying a more complex interface between the domains. Although both depicted vector proteins have simple left–right domain partitioning, Han characters often show more complex arrangements. Examples of this involving 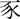 (U+8C55) include 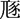 (U+4747), 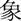 (U+8C61), and 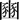 (U+8C73).

**Quaternary structure: multi-character words analogous to multimeric proteins.** Most proteins perform their biological functions as part of protein complexes, which involve either identical protein molecules or different kinds bound together in specific and often symmetrical arrangements (http://www.3Dcomplex.org). Written Chinese provides an analogy here as well. Although the Han characters all originally functioned as stand-alone words, the number of concepts needing words has increased dramatically since the character set became effectively fixed. Instead of inventing new characters, the solution was to combine existing characters to form multi-character words, which are now common. These words are like multi-protein complexes in that their function requires correct arrangement of two or more parts. However, while protein complexes are compound structures, multi-character words are separate structures arranged sequentially. The next section explains how this is implemented in the vector world and considers the implications for functional constraints.

**High-level functions: From sentences to texts, and operons to proteomes.** In both biology and language, the jump from elementary function to useful function brings with it a new level of complexity. Words are elementary semantic units, in that meanings are attached to symbols starting at the word level. But language only becomes useful for communication when word-level meanings are combined to convey more complex meanings. Similarly, although proteins and protein complexes perform low-level functions of biological relevance, organismal capabilities—from survival-enhancing phenotypes all the way up to survival itself—require the coordinated combination of many such functions. Ultimately whole proteomes are coordinated in this way.

**Figure 8:**
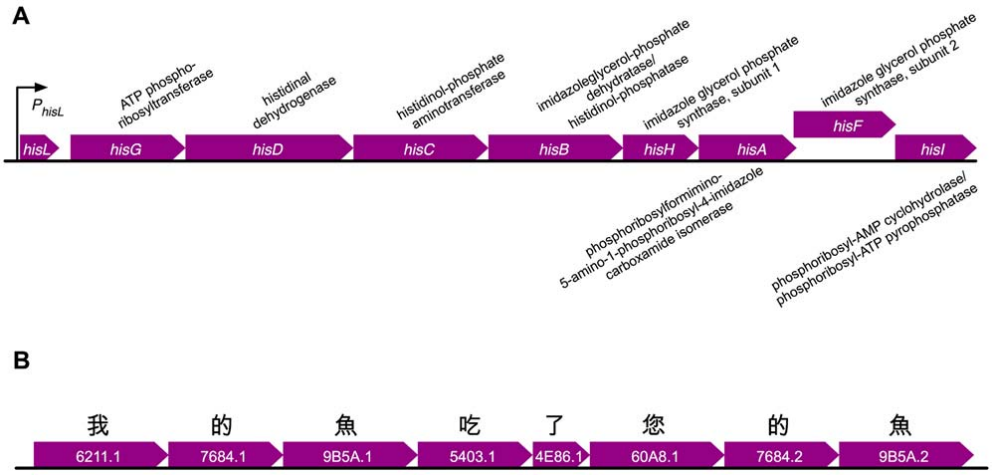
The operon-like structure of vector-world genes encoding a sentence. Gene names shown in white, with functional notation above or below. A) The genetic structure of the histidine operon of *Escherichia coli* (adapted from EcoCyc, http://ecocyc.org). B) The genetic structure of a vector-world gene suite encoding a sentence-level function (see text). Genes are named according to the Unicode number of their function.

In bacterial genomes, the first level of coordination is often achieved by arranging genes in co-regulated blocks called operons (Figure 8A). While there is obvious similarity between genes arranged to produce operon-level functions and words arranged to produce sentence-level functions, gene order appears to be less critical to genome function than word order (syntax) is to linguistic function. If imitation of the protein world were the main objective, the model could be altered to resolve this dissimilarity. But because incorporation of real function is the priority, our approach is instead to allow the vector world to have the properties it naturally inherits from its real linguistic basis. High-level functions in this world are therefore encoded by arranging genes according to the rules of syntax (Figure 8B).

Although these rules result in tighter gene-order constraints in the vector world, this appears to be offset by the absence of two substantial protein constraints. One of these is the requirement, discussed above, for many proteins to form specific multi-protein complexes. Some idea of the difficulty of achieving one specific pair-wise association in these complexes can be had by estimating the number non-productive alternatives that must be avoided. Because that number is quite large (being the number of different surfaces of any kind that compete for interaction), it can easily exceed the number of genes in a bacterial genome. Consequently, even highly stringent constraints on gene order in the vector world are apt to be less restrictive than the constraints of quaternary structure in the protein world.

**Figure 9:**
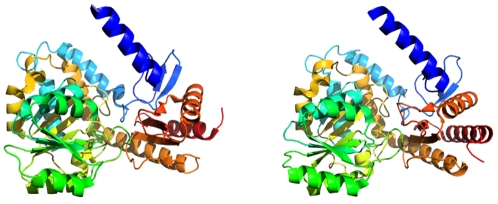
Functional specificity of real proteins depends upon atomic-level details. The products of the *bioF* and *kbl* genes of *E. coli* are virtually indistinguishable at the fold level, but the structural differences produce different functions. Left: BioF monomer (PDB 1DJE), which functions as a dimer in biotin biosynthesis. Right: Kbl monomer (1FC4), which functions as a dimer in threonine degradation.

The second missing constraint in the vector world has to do with specificity of function. Figure 9 shows two proteins that are considered to have the same structure for the purposes of structural classification (e.g., the SCOP classification places them in the same family: http://scop.mrc-lmb.cam.ac.uk/scop/data/scop.b.d.jc.b.f.html). But structural classification necessarily ignores details of structure, focusing instead on secondary structure content and arrangement, and overall chain topology. At the atomic level of active-site structure, where function is determined, these proteins differ decisively. Neither will substitute for the other, and no simple change of just a few amino acids appears to be capable of converting one function to the other (A. Gauger and D. Axe—manuscript in preparation).

Written language behaves differently, as Figure 8B illustrates. The depicted group of genes encode vector proteins that mean: *My fish has eaten your fish*. Notice that the final two gene functions (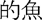) are identical to the second and third. These character pairs are a possessive suffix followed by the symbol for fish, indicating in both instances that the fish referred to belongs to the person just mentioned. The symbols are of course completely interchangeable, but the things they refer to—the fish—are not. In other words, the interpretations of the two instances of 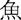 in this sentence differ, even though the symbols are identical. The different meanings result not from structural differences but from syntax—from the different contexts in which the symbols appear. So, what atomic-level structure does for real proteins (provide specific function) syntax does for characters and therefore for vector proteins.

What this facilitates in the vector world is gene recruitment, the process of duplication and functional conversion thought to explain paralogous proteins [20]. In the vector world, genes serving existing functions can produce new high level functions (phrases, sentences, etc.) simply by appropriate side-by-side arrangement of duplicates. Although the protein world does not always require arrangement of this kind for a new high level function (metabolic pathway, molecular machine, etc.) to be formed, the structural reconfiguration of binding surfaces and active sites that it does require appears to be more demanding.

#### Functional proficiency and fitness in the vector world

As indicated, many aspects of the relationship between structure and function in Chinese writing are real-world facts (often complicated ones), with no need for special treatment in the vector world. One aspect that does call for special treatment, though, is legibility—how well written characters conform to the expectations of readers. This has both a qualitative aspect—*What character does this resemble?*—and a quantitative aspect—*How close is the resemblance?* The conventions of Chinese writing provide a qualitative framework for answering the first question, but quantitative answers for the second will be needed for calculating the functional proficiency of vector proteins. This will require both precise standardization of character forms and a precise measure of resemblance, which in turn requires a precise definition of resemblance.

Since human reading cannot be characterized with the required precision, what is needed is a mathematical treatment of resemblance that shows reasonable correspondence with human perception. This will necessarily be much simpler than human character recognition, but to the extent that the human process amounts to an assessment of geometric likeness, we can expect a mathematical assessment of geometric likeness to provide a plausible mapping of structure to functional proficiency. If this is achieved, we will have a mathematical model that defines functional proficiency in the vector world in a way that ties it to something intelligible—legibility (as an aside, existing character-recognition algorithms were found unsuitable because they rely on features that correlate with intended character forms rather than define them, which works when it can be assumed that all forms analyzed are legitimate, but not when structural legitimacy is a point in question).

**Figure 10:**
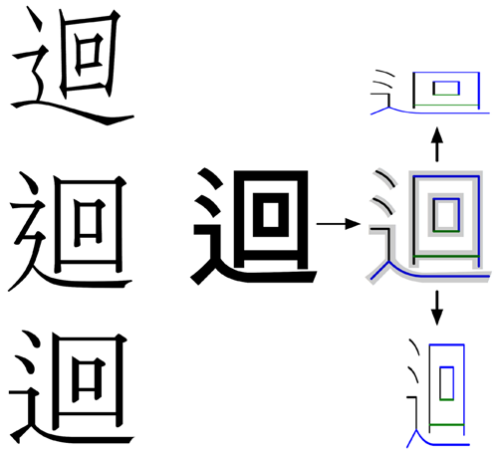
Building archetypes for Han characters. Left: U+8FF4 shown in fonts STFangSong, LiSong Pro, and MS Mincho (top to bottom). Arial Unicode (center) is the chosen standard for archetypes, which are scaleable geometric specifications (right; see text).

**Han archetypes.** Asian fonts provide a starting point for specifying ideal forms for Han characters, which we refer to as Han *archetypes*. However, because fonts show considerable variation in stroke styles and, in extreme cases, even in stroke composition (Figure 10), it is necessary to designate one font as the standard. The primary considerations here are geometric simplicity, widespread availability, and coverage of the traditional character forms. Arial Unicode is most suitable in these respects and has therefore been adopted as the standard.

In terms of structure, characters are simply strokes of particular shapes arranged in a particular way. Han archetypes reflect this by restricting specifications to these structural fundamentals. In particular, conventions of writing technique—the order and direction of stroke formation—are not included. Archetypes are based on line representations of the Arial Unicode forms with individuated strokes (Figure 10). The shape specification for a stroke consists of two or more points designating the ends of the line or curve segments that form the stroke, along with one Bézier control point for each curve segment.

As discussed above (Fold organization), many of the Han characters are built from significant components which may themselves function as stand-alone characters. Because component recognition is an important part of human character recognition (and this mirrors the component-like structure of many real proteins) we include component definitions in archetype specifications. This is done by grouping strokes according to components (if any). Because the aspect ratio of character components is commonly altered in the formation of compound characters (see Figure 2, top), the vector world allows arbitrary rescaling of archetypes with variable aspect ratio (Figure 10). In addition to stroke groupings and shape specifications, a complete archetype specifies stroke placement and any constraints on contacts between strokes (see Text S1 for details).

**Mathematical Model.** If the geometric likeness of a vector protein (working form) to a specified Han archetype can be characterized by a set of separable error metrics *ε*_1_, *ε*_2_, …*ε_n_* having uniformly multiplicative effects, the combined effect on functional proficiency would be described by a decay function of the form:
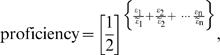
(1)where the constants *ε ~_i_* are set to values that reflect the relative influence of each *ε_i_* on legibility. Since the objective here is to define a proficiency function that captures the key aspects of structural correspondence rather than to model the actual process of human perception, we have chosen this simple form as our basis. We use twelve error metrics to characterize geometric likeness in terms of the shape, size, placement, and connectivity of strokes, the size and placement of domain-like groups of strokes, and the presence of any overall flaws like extraneous marks or gaps within strokes (see Text S1 for details). As shown below, this way of mapping structure to functional proficiency does provide reasonable correspondence both with human perception and with the protein world.

**Figure 11:**
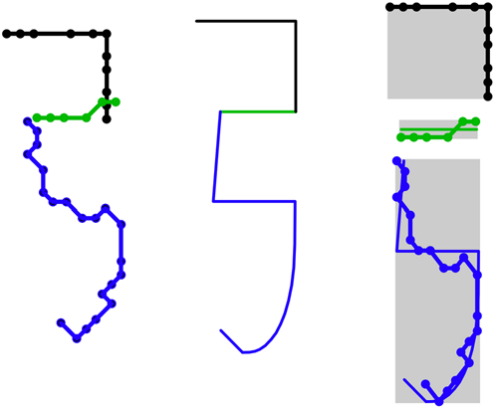
Assessing shape distortion of vector strokes by comparing with ideal forms. Colors differentiate the three strokes forming a component of 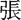 (U+5F35). Dots show vector boundaries. Left: strokes from a vector protein with a proficiency score of 0.4 (shown in Figure 12). Middle: the ideal structure specified by the archetype. Right: scaled vector strokes laid over their archetype forms, with bounding rectangles shaded. Shape distortion is assessed for each stroke individually, the top stroke in this example having no distortion.

Calculation of a proficiency score begins by scaling vector strokes individually such that their widths and heights match those of the corresponding archetype strokes (Figure 11). Scaled vector strokes and their archetypes are then overlaid in order to quantify shape distortion. By experimenting with root mean square deviation (RMSD) as a shape distortion metric, we found that archetype strokes consisting exclusively of horizontal and vertical lines allowed much less conformational freedom in vector proteins than strokes with curves did. Maximum deviation was then tested and found to provide more uniform conformational freedom along with comparably good representation of readability. We therefore chose this metric for quantifying shape distortion (see Text S1 for details of calculation).

To reflect the importance of components within compound characters, group proficiencies are calculated separately for each grouping of vector strokes defined by the archetype. Group proficiencies reflect not only the average maximum deviation of the contained vector strokes, but also any inconsistency in their scaling or placement. Equation 1 is applied with an *ε_i_* metric representing each of these errors. The proficiency for the whole character is then calculated from the group-level proficiencies, taking further account of any errors in the structural arrangement of groups within the character. Equation 1 applies again at this level, but instead of including errors internal to all groups, only those pertaining to the least proficient group are included. This “weakest-link” approach reflects that fact that the whole function results from different components performing their own sub-functions, such that overall proficiency is most readily achieved by comparably proficient sub-components. Similar reasoning applies in the case of a multi-character message. Since each character performs a separate sub-task, and the overall task amounts to adequate performance of each of these sub-tasks, the functional proficiency of a message is simply the lowest proficiency of its constituent characters (Zeldovich and co-workers likewise used the weakest-link approach, as described in reference 8).

**Figure 12:**
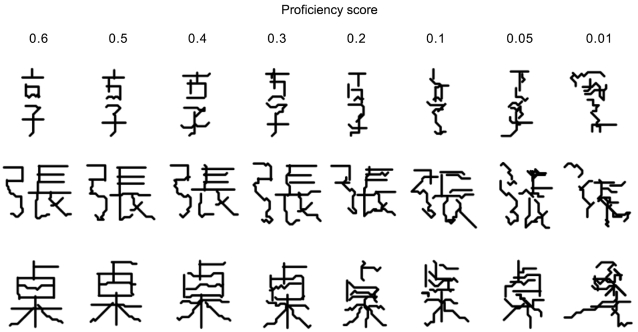
Qualitative correlation between functional proficiency of vector proteins and their legibility. Initial genes encoding 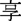 (U+4EAB), 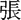 (U+5F35), and 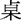 (U+684C) were generated with *Inscribe* and processed with *Stylus* (see *Software*). Initial proficiencies were above 0.6. Selection thresholds ran from 0.60 to 0.10 in steps of −0.05, followed by thresholds of 0.07, 0.05, 0.03, and 0.01. At each threshold, 1000 non-synonymous base substitutions were accumulated. Vector proteins shown are representative of the distortion seen at the indicated scores.

A simple way to see whether proficiency scores computed in this way show reasonable correspondence with legibility is to subject highly proficient genes to random point mutations, accepting only those that leave the proficiency above a specified threshold. Because mutations tend to be disruptive, propagated lines evolved in this way tend to hover just above the threshold. So, by lowering the threshold in small steps, we can produce a long line of descent showing gradual decline in proficiency. The software and methods for doing this kind of experiment will be introduced next. Here we aim merely to verify the intended qualitative connection between proficiency and legibility. Figure 12 shows snapshots at various stages of decline in three unrelated lines. Legibility shows a similar decline with decreasing proficiency in all cases, indicating that calculated proficiencies correlate reasonably well with actual function.

Fitness is treated by a simple extension of the proficiency model outlined above. Being a property of whole organisms or whole genomes, fitness involves not just how well necessary functions are performed (proficiency) but also the *cost* of performing them. The vector world bases cost on usage of the two monomer types: DNA bases and vectors. A gene carries a cost calculated by multiplying its length by a per-base cost, and adding to this the total length of its encoded vector path times a per-unit vector cost. The fitness of a genome is then calculated by dividing the lowest proficiency of its necessary functions by the total cost of its genes (see Text S1 for details).

### Software

Two applications have been developed for designing and performing experiments in the vector world. Brief overviews of these are provided here. For detailed descriptions and software download, see the project site (http://sourceforge.net/projects/biologicstylus/).

#### Inscribe—a tool for building archetypes and genes

**Figure 13:**
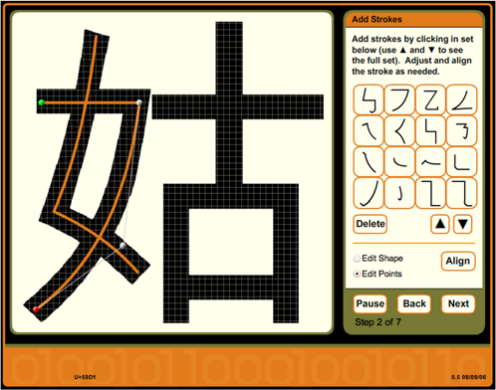
*Inscribe* screenshot, showing archetype construction for 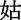 (U+59D1).

*Inscribe* is a Flash application that runs within standard web browsers. One of its functions is to facilitate the construction of Han archetypes. It does this by displaying the enlarged Arial Unicode character on a grid, enabling the user to trace the path of each stroke by selecting pre-defined stroke forms from a palette and adjusting shapes and sizes as needed (Figure 13). On-screen instructions guide the user through subsequent steps for specifying stroke groups and constraints on stroke-to-stroke contacts.

Secondly, *Inscribe* generates genes corresponding to a specified archetype, allowing the user to control gene size along with the order and direction of strokes in the encoded vector protein. The algorithm used by *Inscribe* to trace an archetype produces genes that encode highly regular vector proteins. By subjecting these to extensive mutation and selection (using *Stylus*—see below), genes encoding paths with realistic irregularity (i.e., irregularity that is consistent with a real evolutionary history) are easily produced. Genes naturalized in this way serve as a starting point for genetic experimentation.

#### Stylus—a system for line-of-descent experiments in the vector world

**Figure 14:**
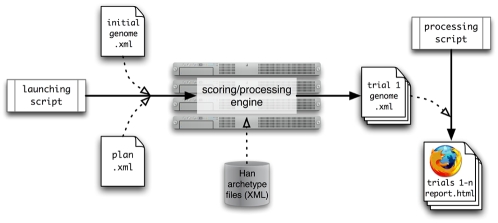
*Stylus* process overview. The system design accommodates either one-at-a-time processing or batch processing on a grid.

*Stylus* provides a full implementation of the vector world described here. It consists of a binary engine for rapid scoring and processing of genes, along with scripts for launching experimental plans and processing output (Figure 14). *Stylus* plans specify the conditions under which an initial gene is mutated and propagated. A versatile scripting vocabulary has been developed for this purpose, enabling the user to apply an assortment of mutations (point changes, block changes, insertions, deletions, duplications, or transpositions) with specified likelihoods. Further, the ability to target separate mutation profiles to any number of regions along the gene allows emulation of complex mutation phenomena like hotspots.

*Stylus* processes a gene serially through any number of mutational trials. Completion of a trial occurs when selection conditions specified in the plan are met. Trials therefore correspond not to generations but to consecutive allele replacement events in a propagated cell line (where many generations may pass between these replacements). Again, the scripting vocabulary for building plans enables selection conditions to be specified in a variety of ways, including a single fitness or proficiency threshold, probabilistic distributions of multiple thresholds, or relative thresholds like: advance trial if an attempted mutation produces a fitness above 98% of the current fitness (from most recently completed trial).

**Figure 15:**
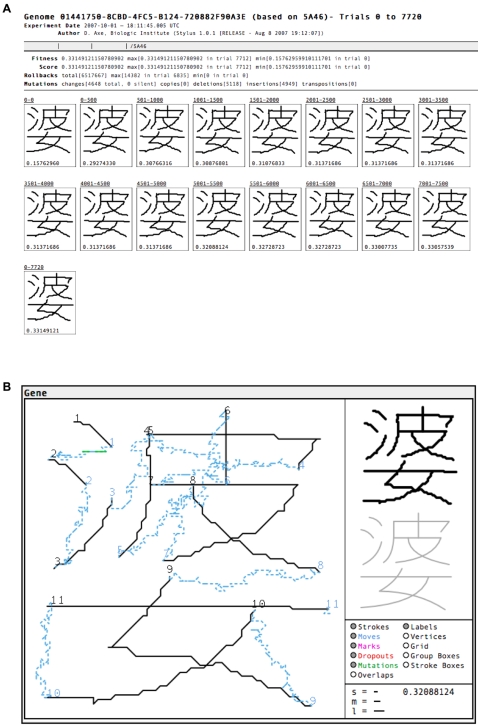
*Stylus* report screenshots. A) Summary page, showing vector proteins at regular intervals along a line of descent. B) Structural section of a detail page for a single trial. Other sections on the same page provide details of fitness and proficiency scores, mutation history, and gene/protein sequences.

The architecture of *Stylus* anticipates two future enhancements. One of these is genome-scale processing, where the engine operates directly on genomes consisting of many genes. While the vector-world model readily extends to this scale, *Stylus* 1.0 (the initial release version) operates on single genes. The second anticipated enhancement is a web-hosted service that would enable users to design and run experiments without having to understand technical aspects of *Stylus* operation. Report generation, among other things, is designed to facilitate this. While running an experimental plan, the *Stylus* engine writes output at specified intervals in the form of XML files named by trial number. These data files provide detailed information about the current gene, the vector protein it encodes, and the succession of mutation attempts that preceded it. A Python script uses this information to generate a user-friendly interactive report accessed through a standard web browser (Firefox 2.0 or above, Safari 3.0 or above, Internet Explorer 6 or above). Reports begin with a summary page showing vector proteins at the specified intervals (Figure 15A). Clicking above any of these images loads an interactive page with visually intuitive presentations of structure, scoring, mutation, and sequence details for that trial (Figure 15B).

### Analysis—Using *Stylus* to examine real problems by analogy

Because of its vast size, protein sequence space allows very limited exploration, whether by experiment or by computational modeling. An advantage of modeling, though, is that the experimenter has more freedom to use the limited sampling resources in the most productive way. This opens a number of avenues for addressing important questions that are not easily addressed in the laboratory. How modular are structural motifs? What is the simplest route to new protein folds? How far do structures drift when sequences drift? How do periods of relaxed selection affect neutral evolution? How would protein evolution differ if the genetic code differed? How might the genetic code evolve? Real-world questions like these have direct equivalents in the vector world, where *Stylus* enables them to be addressed.

Like all computational approaches, *Stylus* also offers the considerable advantage of comprehensive information. Because all aspects of an experiment may be examined in detail and the same experiment may be performed repeatedly with realistic random variation, histories and statistical relationships can be characterized with precision. Consequently, what can only be inferred from real-world data can often be demonstrated with vector-world data.

To illustrate how experiments are performed with *Stylus*, and to further demonstrate the strength of the vector-world analogy, we will look at two simple examples.

#### Generating homologous sets by near-neutral divergence

As mentioned above (Core Analogy), natural protein sequences can diverge substantially while maintaining their original function. Although sequence divergence leads to structural divergence [21], protein structures retain clear similarity even past the point where sequence similarity becomes hard to detect [22], [23]. Can this basic aspect of neutral drift be replicated in the vector world?

To test this, we generated homologous genes by running several line-of-descent experiments on the same initial gene. The same experimental plan was used each time, with run-to-run variation ensured by specifying different seed values for the random number generator. Most aspects of the experimental design are incorporated in a single nested block within the plan XML file. For the current example, that block looks like this:

01:<step trials = ‘1000000’>
02:<trialConditions>
03:<scoreCondition gene = ‘1’ mode = ‘maintain’>
04:<value value = ‘0.45’ likelihood = ‘1’/>
05:</scoreCondition>
06:<mutationCondition>
07:<mutationsPerAttempt likelihood = ‘1.0’ count = ‘1’/>
08:</mutationCondition>
09:</trialConditions>
10:<change likelihood = ‘0.998’/>
11:<delete likelihood = ‘0.001’ countBases = ‘3’/>
12:<insert likelihood = ‘0.001’ countBases = ‘3’/>
13:</step>

The first line specifies that this experiment consists of a million trials, each trial being a single instance of a mutant allele replacing the previous allele by passing the selection condition. That condition is specified in lines 3 though 5, which require the proficiency score of a passing mutant to be at least 0.45. Plans may use multiple lines in the form of line 4 (with likelihoods adding to 1) to apply more sophisticated probabilistic selection criteria. Line 7 specifies that each attempt involves only a single mutation, though again the structure of *Stylus* allows simultaneous mutations conforming to any frequency distribution. Lines 10 through 12 specify the kinds of mutation that will be applied, along with their likelihoods. In this case, 99.8% of the mutations will be single base substitutions with equal likelihood (the default behavior of change, when no modifiers are specified—*Stylus* allows simultaneous changing of multiple bases, changes to specified sequences, and random changes with specified transition/transversion bias), 0.1% will be codon deletions, and 0.1% will be random codon insertions. By default, the locations will vary randomly (uniform distribution), though specific locations or base ranges can be specified.

Termination conditions (not shown) enable the experiment to stop before all trials are completed. By specifying a maximum of one million *attempts*, we can simulate neutral evolution over a period where a non-functional gene would accrue that many mutations (selection eliminating most of these from a functional gene). For a bacterial genome consisting of about a thousand genes of comparable size, this corresponds roughly to a few hundred million years of evolution (based on a mutation rate of 0.003 per genome per replication [24] and a generation time of 0.001 year [25]).

**Figure 16:**
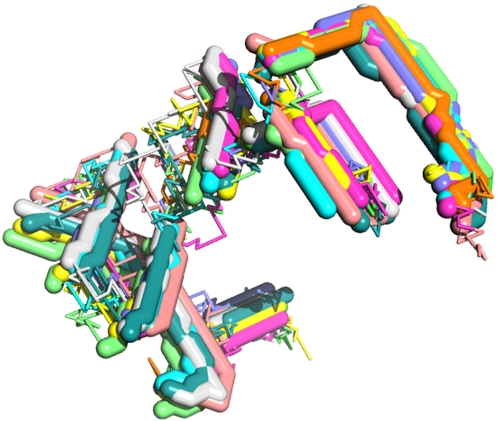
Structural superposition of ten homologous vector proteins having the function of 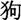 (U+72D7). Structures were aligned by translation in the three coordinate directions without rotation (thereby preserving vector directions).

**Figure 17:**
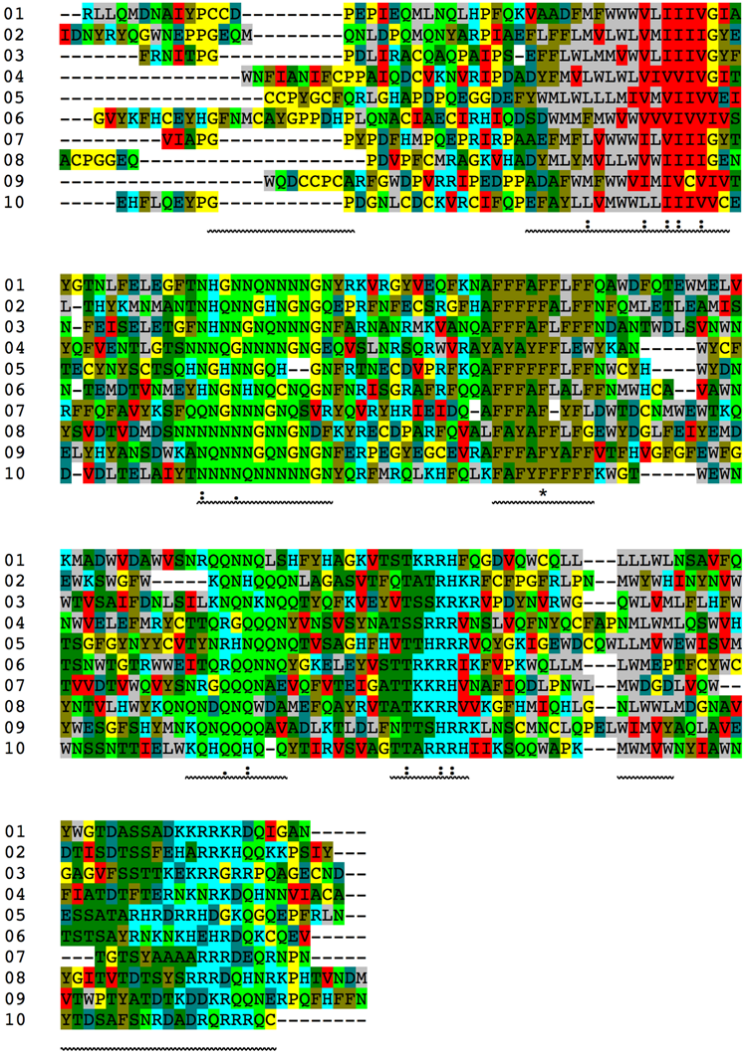
Sequence alignment of ten homologous vector proteins. The vector proteins shown in Figure 16 were aligned with ClustalW [26] without using amino-acid based information (i.e., using the identity matrix for substitution scoring and no protein-based gap penalties). Vectors were assigned single-letter amino acid codes (L = Nos; M = Nom; W = Nol; F = Nes; Y = Nem; A = Eas; S = Eam; T = Eal; D = Ses; E = Sem; H = Sos; K = Som; R = Sol; N = Sws; Q = Swm; G = Wes; C = Wem; P = Wel; V = Nws; I = Nwm) and colored according to direction (No = grey; Ne = gold; Ea = deep green; Se = teal; So = cyan; Sw = bright green; We = yellow; Nw = red). Asterisk indicates position of complete vector conservation (dots are not meaningful, being based on amino-acid similarities). Wavy underlines show stroke locations for sequence 10 (locations vary somewhat among sequences).

Figure 16 shows ten vector proteins produced from a single ancestral gene in this way. Like real proteins, these vector proteins show more structural divergence in regions of irregular structure (moves—corresponding to loops) than in regions of regular structure (strokes—corresponding to secondary structure). Their sequences have diverged to the point where only one vector is fully conserved (Figure 17). With pairwise identities of 22–30% (using stretcherP [27]), these sequences fall within the “twilight zone” of homology detection for real proteins [23]. Still, the aligned sequences show clear similarity when vectors are grouped according to their direction (Figure 17), and like natural sequences they show greater divergence near termini and between regions of regular structure.

Because the vector world captures many aspects of the real protein world, the ability to generate distantly related sequences and structures with complete histories should allow a number of interesting problems to be addressed that cannot be addressed experimentally. For example, are unrelated sequences that have converged on similar structures reliably distinguishable from very distantly related sequences? If so, are some comparison tools better than others at differentiating these two cases?

#### Measuring the functional effects of single substitutions

Although most amino-acid substitutions reduce protein stability and function somewhat [6], [28], single changes usually do not have catastrophic effects [29]. Rather, mild disruption is buffered to some extent by excess structural stability [30], [31]. When this excess is exhausted by cumulative mutation without purifying selection, catastrophic impairment occurs [6].

To see how vector proteins respond to point mutations, we used *Stylus* to generate and score 100,000 random single base substitutions for each of the 10 homologous genes produced in the preceding example. This could have been done in a number of ways. Our approach used a plan that differs from the previous one in three respects: the number of attempts is limited to 100,000; the required proficiency score is raised to 1.0; and the change likelihood (line 10) is raised to 1.0, with removal of other mutations (lines 11 and 12). Requiring perfect proficiency for a trial to be completed causes *Stylus* to run through 100,000 “failed” attempts, the result of each of these attempts being recorded before the mutation is undone and the next applied. Each result is added to the output text file in the form of an XML tag like this:

<attempt description = 

‘scoreCondition failed: Value(0.17407) Threshold(1.0) Mode(maintain)’>

<changed targetIndex = ‘308’ countBases = ‘1’ bases = ‘T’ basesAfter = ‘C’/>

</attempt>,

making it relatively straightforward to extract the desired information.

**Figure 18:**
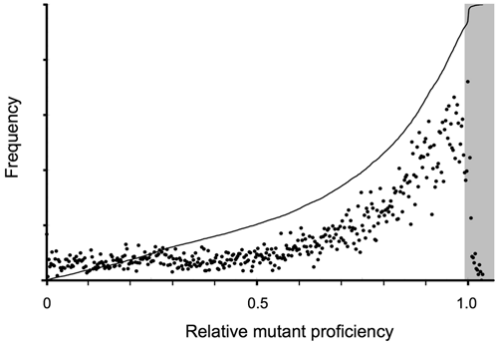
Effects of non-synonymous point mutations on vector protein function. Relative mutant proficiencies were calculated by dividing mutant proficiencies by the pre-mutation proficiency, with the resulting values binned in increments of 0.0025. Points show how many mutations fall into each bin, the vertical scale running from zero to 10,000. The line shows the proportion of mutations (zero to one) with relative proficiencies less than or equal to the value on the horizontal scale. The point representing true neutral substitutions (30,203 mutations with relative proficiency = 1) is above the range shown.

Combining the data from all ten output files (1 million total mutations), we find that just under 75% of the base changes were non-synonymous (causing a vector substitution). About 3.7% of these non-synonymous changes cause a ten-fold or greater drop in proficiency (Figure 18), similar to the proportion of amino-acid substitutions found to inactivate a bacterial ribonuclease (5% [29]). In the vector proteins, only a tiny fraction (0.4%) of the changes are so disruptive that proficiencies could not be calculated (this occurs when strokes in the vector protein cannot be mapped to strokes in the archetype—for example, when two strokes merge into one). The most common effect is a modest proficiency reduction of 0–5% (Figure 18). Again, this is consistent with the behavior of real proteins, which tolerate conservative substitutions in substantial numbers before function is lost completely [6], [32]. It also accounts for the neutral divergence demonstrated above. The shaded region in Figure 18 indicates the neutral zone, where the frequency-averaged effect on proficiency is zero. Point mutations falling within this zone, amounting to about 8% of the non-synonymous total, can accumulate indefinitely without net loss of function. This percentage compares well with the actual ratio of non-synonymous substitutions to synonymous substitutions in bacterial genes [33].

## Discussion

Whether conclusions from artificial models apply to real proteins is an issue common to all models. Useful models offset this limitation in at least two ways. First, what they lack in biological realism they make up for in conceptual precision and tractability. They are real conceptual systems that lend themselves to investigation, even though they differ from the natural systems they emulate. And second, while they never bridge the gap to nature, they can aim for a degree of similarity that makes comparison of the two systems interesting and informative.

Lattice models have long achieved this in the study of protein folding [34], [35]. Because lattice proteins have real sequences that map to a space of real structures, they can be used to define and study real, albeit conceptual, folding problems. But because they lack real function, it is difficult to see how this approach can accomplish for protein evolution what it has accomplished for protein folding. Where there is no real function, there is no real functional problem being studied, even conceptually.

The model described here offers a solution to this. Because legible characters perform real functions, vector proteins occupy positions in a structure space that has a genuine connection to function. Quantitative description of this connection requires simplifying approximations, but this is a universal aspect of modeling complex phenomena. The primary advantage of the vector-world model over lattice models is that it has a real structure–function relationship to simplify—one that also shows the multi-faceted, multi-layered complexity characteristic of biological systems (Table 1).

**Table 1:** Summary of Correspondence Between Real World and Vector World

Corresponding pairs		Comments		
Vector Proteins	Natural Proteins	Primary similarities	Primary differences	Implications
Existing analog: *Han characters* with their associated *meanings*	*Protein structures* with their associated *functions*	Real-world mappings of structure to function. Real natural histories. Similar set sizes.	Characters are 2D; proteins are 3D. Characters are geometric; proteins are physical.	Opens possibility of constructing an artificial protein model around a real and tractable structure–function relationship. Static nature of written forms precludes dynamic folding model.
Existing analog: *Legibility* (how well a written character performs its function)	*Activity* (how well a folded protein performs its function)	As real-world phenomena, both carry real, complex constraints.	Legibility is observer dependent.	Opens possibility of evolutionary simulation under realistic functional constraints, with the limitation that numerical approximation will be required.
Constructed analog: *The 20 vectors*	*The 20 amino acids*	Multiple structural aspects. Each monomer is distinct.	Vectors have only two properties, whereas amino acids have many.	Space of structural possibilities for protein-length polymers is vast for both worlds.
Constructed analog: *Vector-world genetic code*	*Natural genetic code*	64 codons mapped to 20 monomers (plus start and stop). Third-position degeneracy.	More uniform representation of vectors (2 to 4 codons). Synonymous vector codons are precisely equivalent.	Synonymous substitution rate is precisely proportional to incident mutation rate in vector world.
Constructed analog: *Vector genes*	*Bacterial genes*	Identical open-reading-frame structure. Similar typical lengths.	Vector gene expression has no dynamic aspect.	Full analogy to static aspects of bacterial genetics, though not suitable for studying genetic regulation.
Constructed analog: *Vector proteins*	*Natural proteins*	Polymers of similar length that perform specific functions by means of well-defined structures.	Vector proteins have no folding process; no analog of active sites.	Rich sequence-to-structure analogy, strength being static structure not structural dynamics or enzyme kinetics.
Constructed analog: *Vector protein structures*	*Tertiary backbone structures*	Like real proteins, vector proteins have fold-like structural hierarchy, topological complexity, and a highly many-to-one mapping of structure to function.	Vector paths are static 2D structures. Protein backbones form dynamic 3D structures.	Rich structure-to-function analogy, though limited to static aspects.
Constructed analog: *Coherent vector regions*	*Regular (secondary) structure*	Aspects of local chain geometry. More critical (than incoherent/irregular) for forming whole structure. Small breaks in regular structure may be tolerated.	Local vector structure is autonomous, whereas local protein structure forms cooperatively.	Autonomy of local vector structure may simplify modular assembly of genes encoding new structures.
Constructed analog: *Incoherent vector regions*	*Irregular structure*	Aspects of local chain geometry. Both connect units of regular structure. Single substitutions can induce regular structure.	Irregular ‘loops’ are often involved in forming natural active sites.	Both worlds show interplay between regular and irregular structure, where boundaries tend to shift upon mutation.
Mainly existing analog: *Vector strokes and stroke motifs*	*Structural motifs*	Basic structural components found in all structures. Many show topological variation.	Vector components are structurally autonomous, whereas protein components form cooperatively.	Autonomy of sub-structures in vector world may simplify modular assembly of genes encoding new structures.
Existing analog: *Sub-functions performed by groups of vector strokes (vector domains)*	*Sub-functions performed by structural domains*	In both worlds, functionally significant parts combine to produce compound functions.	Vector domains are structurally autonomous, which may or may not be the case for protein domains.	Autonomy of functional sub-structures in vector world may simplify modular assembly of genes encoding new functions.
Mainly existing analog: *Gene order*	*Genomic and proteomic organization*	Genome organization affects high-level function in both worlds.	Vector gene order is constrained by rules of syntax. Bacterial gene interactions are less dependent on gene order.	Opens possibility of evolutionary simulation within genome-level functional constraints, though the form of these constraints is simpler in the vector world.
Mainly existing analog: *Multi-protein words*	*Multi-protein complexes*	In both worlds, many functions require two or more proteins to come together.	Natural protein complexes are compound structures, whereas vector protein complexes follow from gene order.	Combining proteins to form a compound function may be simpler in the vector world, requiring juxtaposition of genes rather than construction of specific binding interfaces.
Mainly existing analog: *Multi-protein messages*	*Multi-protein systems*	Protein-level functions may be combined to produce higher levels of function in both worlds.	High-level biological functions often require regulated expression and transport, in addition to complex formation.	Construction of protein systems with high-level function may be simpler in the vector world, requiring only correct gene order.

Other advantages come from the more life-like sequence and structure spaces of the vector world. The highly restricted spaces of most lattice models often allow complete enumeration of the possibilities. As helpful as this is for examining things like global free-energy minima, it is very unlike the real world of proteins. Because the vast size of the real spaces is a major part of the problem that evolutionary theories must grapple with, models that capture this close the gap to nature in a significant respect. Furthermore, since these theories are largely probabilistic, exhaustive enumeration is unnecessary. Instead, the highly efficient sampling that can be achieved with computational models provides measurements that are both meaningful and comparable to real-world measurements.

As a full-featured implementation of the vector world, *Stylus* enables challenging evolutionary problems to be tackled in a model world having a level of realism that may allow informative comparison with biology. Despite the life-size genes and proteins it processes, computational performance is sufficient for useful results to be obtained with modest resources (e.g., the line-of-descent runs to produce sequences in Figure 17 used under 5 minutes of cpu time each on 3 GHz Intel Xeon processors). And because vector-world genes and proteins are described by the same text-file formats used for real genes and proteins, vector-world studies benefit from numerous existing bioinformatics tools. Finally, because the vector world is built on a structure–function relationship that is not only real but also visually intuitive, *Stylus* offers the attractive possibility of clarifying complex problems.

## Supporting Information

Text S1 This document gives details of the mathematical algorithm used to calculate proficiency and fitness scores.
(1.43 MB PDF)
