# Supplementary material for: Stylus: A System for Evolutionary Experimentation Based on a Protein/Proteome Model with Non-Arbitrary Functional Constraints
Source: PLoS One. 2008 Jun 4;3(6):e2246. doi: 10.1371/journal.pone.0002246 (PMC2405935; doi:10.1371/journal.pone.0002246)
Supplement: Text S1 — This document gives details of the mathematical algorithm used to calculate proficiency and fitness scores. (1.43 MB PDF) [file pone.0002246.s001.pdf]

Stylus Scoring Algorithm

Scoring refers to calculation of the functional proficiency of a vector-world gene with respect to a specified Han character. It involves calculations performed at four levels: stroke level, stroke-group level, gene/protein level, and genome/proteome level. Up to and including the gene/protein level, the objective is to quantify how well the vector protein encoded by the gene conforms to the Han archetype. At the genome/proteome level scoring becomes analogous to fitness, being dependent both on functional proficiencies and on resource consumption.

The degree of resemblance between the vector protein and the archetype is evaluated first at the stroke level, then at the group level, and finally at the gene/protein level. All deviations involving measurements of length are performed in the coordinate system of the archetype. Consequently, it is necessary to transform vector-protein coordinates to map points onto the Han archetype. Because the scaling involved at each level may depend on scaling at higher levels (explained below), it is necessary to start with evaluation of scale factors up to the gene/protein level.

1. Assigning scale factors to vector proteins and their components

1.1. Vector-protein strokes are considered to have an *inherent* scale in the x dimension if and only if both they and the corresponding archetype stroke have non-zero width. The corresponding condition (non-zero height) applies in the y dimension.

1.2. A vector-protein stroke with an inherent x scale is assigned the following stroke-level x-scale factor:

$$sx_S = W_S / w_S,$$

where W_S and w_S are the widths of the boxes enclosing the archetype and vector versions of the stroke in their respective coordinates.

The y dimension is treated equivalently ($x \to y; W \to H; w \to h$).

1.3. Within the vector protein, a stroke group containing at least one stroke with an inherent x scale is itself considered to have an inherent x scale. The x-scale factor assigned to such a group is a width-weighted mean of its stroke-level scale factors:

$$sx_G = \frac{\sum_{i=1}^{n} \left(W_{Si} \cdot sx_{Si} \right)}{\sum_{i=1}^{n} W_{Si}},$$

where the summations are over all contained strokes with an inherent x scale.

The y dimension is treated equivalently ($x \to y; W \to H$).

1.4. A vector protein containing at least one stroke group with an inherent x scale is itself considered to have an inherent x scale. The x-scale factor assigned to such a protein is a width-weighted mean of its group-level scale factors:

$$sx_P = \frac{\sum_{i=1}^{n} \left(W_{Gi} \cdot sx_{Gi} \right)}{\sum_{i=1}^{n} W_{Gi}},$$

where the summations are over all contained stroke groups with an inherent x scale, W_{Gi} being the width of the box enclosing the ith such group in the archetype.

The y dimension is treated equivalently ($x \to y; W \to H$).

1.5. With inherent scale assignments now complete, all objects lacking inherent scales in either dimension are *assigned* scale factors as follows:

1.5.1. If the vector protein lacks an inherent x or y scale, the missing scale factor is set equal to the other one (i.e., $sx_P = sy_P$).

1.5.2. Any stroke group in the vector protein that lacks an inherent x or y scale is then assigned the corresponding protein level value (which may itself have been assigned).

1.5.3. Finally, stroke in the vector protein lacking an inherent x or y scale is assigned the corresponding stroke-group level value (which may itself have been assigned).

2. Object centers—definitions and notation

2.1. $\dot{X}_S$ denotes the x coordinate of the center point of the box enclosing an archetype stroke (in archetype coordinates). Likewise for $\dot{Y}_S$.

2.2. $\dot{x}_S$ denotes the x coordinate of the center point of the box enclosing a vector-protein stroke (in vector coordinates). Likewise for $\dot{y}_S$.

2.3. $\dot{X}_G$ denotes the x coordinate of the weighted center of a stroke group in the archetype, calculated as:

$$\dot{X}_G = \frac{\sum_{i=1}^{n} L_{Si} \cdot \dot{X}_{Si}}{\sum_{i=1}^{n} L_{Si}}, \text{ or equivalently: } \dot{X}_G = \frac{\sum_{i=1}^{n} L_{Si} \cdot \dot{X}_{Si}}{L_G}$$

where the summations are over all contained strokes, L_{Si} denotes the path length of a stroke within the archetype stroke group, and L_G denotes the total path lengths of all strokes in the archetype stroke group.

The *y* dimension is treated equivalently ($X \to Y$).

2.4. $\dot{x}_G$ denotes the *x* coordinate of the weighted center of a stroke group in the vector protein, calculated as:

$$\dot{x}_G = \frac{\sum_{i=1}^{n} L_{Si} \cdot \dot{x}_{Si}}{\sum_{i=1}^{n} L_{Si}}, \text{ or equivalently: } \dot{x}_G = \frac{\sum_{i=1}^{n} L_{Si} \cdot \dot{x}_{Si}}{L_G}$$

where summations are over all contained strokes.

The *y* dimension is treated equivalently ($x \to y$).

2.5. $\dot{X}_P$ denotes the *x* coordinate of the weighted center of the full archetype, calculated as:

$$\dot{X}_P = \frac{\sum_{i=1}^{n} L_{Gi} \cdot \dot{X}_{Gi}}{\sum_{i=1}^{n} L_{Gi}}, \text{ or equivalently: } \dot{X}_P = \frac{\sum_{i=1}^{n} L_{Gi} \cdot \dot{X}_{Gi}}{L_P}$$

where the summations are over all contained groups, L_{Gi} denotes the total path length of a stroke group within the archetype, and L_P denotes the total path lengths of all strokes in the archetype.

The *y* dimension is treated equivalently ($X \to Y$).

2.6. $\dot{x}_P$ denotes the *x* coordinate of the weighted center of the vector protein, calculated as:

$$\dot{x}_P = \frac{\sum_{i=1}^{n} L_{Gi} \cdot \dot{x}_{Gi}}{\sum_{i=1}^{n} L_{Gi}}, \text{ or equivalently: } \dot{x}_P = \frac{\sum_{i=1}^{n} L_{Gi} \cdot \dot{x}_{Gi}}{L_P}$$

where summations are over all contained groups.

The *y* dimension is treated equivalently ($x \to y$).

3. Translational offsets—definitions and notation

The following translational offsets are used in coordinate transformations (for mapping vector-protein points onto the archetype):

3.1. *Stroke-scaled stroke offsets*: $dx_S = \dot{X}_S - sx_S \cdot \dot{x}_S$; $dy_S = \dot{Y}_S - sy_S \cdot \dot{y}_S$

3.2. *Group-scaled stroke offsets*: $dx_{(G)S} = \dot{X}_S - sx_G \cdot \dot{x}_S$; $dy_{(G)S} = \dot{Y}_S - sy_G \cdot \dot{y}_S$

3.3. *Group-scaled group offsets*: $dx_G = \dot{X}_G - sx_G \cdot \dot{x}_G$; $dy_G = \dot{Y}_G - sy_G \cdot \dot{y}_G$

3.4. *Protein-scaled group offsets*: $dx_{(P)G} = \dot{X}_G - sx_P \cdot \dot{x}_G$; $dy_{(P)G} = \dot{Y}_G - sy_P \cdot \dot{y}_G$

3.5. *Protein-scaled protein offsets*: $dx_P = \dot{X}_P - sx_P \cdot \dot{x}_P$; $dy_P = \dot{Y}_P - sy_P \cdot \dot{y}_P$

4. Stroke-level calculations

4.1. The *maximum deviation* between a vector-protein stroke and the corresponding archetype stroke is assessed by using stroke-level scale factors and offsets to map the vector-protein stroke onto the archetype stroke, and then measuring the distance between corresponding points on the two versions of the stroke. Points are considered to correspond if they mark equal fractional progress along their respective stroke paths. So the following relation is used to identify corresponding points on the two paths:

$$\phi_i \equiv \frac{\text{archetype path length to point } (X_i,\ Y_i)}{\text{path length of archetype stroke}} = \frac{\text{path length of transformed vector-protein stroke to point } (\hat{x}_i,\ \hat{y}_i)}{\text{path length of transformed vector-protein stroke}}$$

where $\hat{x}_i$ and $\hat{y}_i$ are the transformed coordinates of the ith comparison point in the vector-protein stroke:

$\hat{x}_i = sx_S \cdot x_i + dx_S$, and $\hat{y}_i = sy_S \cdot y_i + dy_S$.

The maximum deviation for a given stroke (in archetype coordinates) is then:

$$\delta = Max\left\{\sqrt{(X_1-\hat{x}_1)^2+(Y_1-\hat{y}_1)^2},\ldots,\sqrt{(X_n-\hat{x}_n)^2+(Y_n-\hat{y}_n)^2}\right\}$$

where the n points for comparison consist of all segment endpoints in the archetype stroke along with all transformed vector endpoints.

4.2. *Excess path length* is calculated for strokes as follows:

$$e = \mathrm{Max}\left\{0,\ \left[\sum_{i=1}^{m-1}\sqrt{(\hat{x}_{i+1}-\hat{x}_i)^2+(\hat{y}_{i+1}-\hat{y}_i)^2}\right]-\left[\sum_{i=1}^{n-1}\sqrt{(X_{i+1}-X_i)^2+(Y_{i+1}-Y_i)^2}\right]\right\},$$

where the first summation is over transformed vector endpoints in the stroke (m endpoints in total) and the second is over archetype segment endpoints (totaling n).

5. Calculating proficiencies for stroke groups

5.1. The proficiencies with which stroke groups in a vector protein perform the sub-functions of the corresponding groups in the archetype are calculated according to:

$$\Pi_G = \left[\frac{1}{2}\right]^{\left\{\frac{\varepsilon_{Gs}}{\tilde{\varepsilon}_{Gs}}+\frac{\varepsilon_{Gp}}{\tilde{\varepsilon}_{Gp}}+\frac{\varepsilon_{\delta}}{\tilde{\varepsilon}_{\delta}}+\frac{\varepsilon_{e}}{\tilde{\varepsilon}_{e}}+\frac{\varepsilon_{Gb}}{\tilde{\varepsilon}_{Gb}}+\frac{\varepsilon_{Gc}}{\tilde{\varepsilon}_{Gc}}+\frac{\varepsilon_{d}}{\tilde{\varepsilon}_{d}}\right\}}$$

where each ε_i is dimensionless error measure (defined below), and $\tilde{\varepsilon}_i$ is a constant with a value that reflects the relative influence of ε_i on legibility.

The dimensionless error measures are defined as follows:

5.2. Inconsistency of stroke scaling within group

$$\varepsilon_{Gs} = \frac{1}{sx_G}\sqrt{\frac{\sum_{i=1}^{m} W_{Si}\left(sx_G - sx_{Si}\right)^2}{\sum_{i=1}^{m} W_{Si}}} + \frac{1}{sy_G}\sqrt{\frac{\sum_{i=1}^{n} H_{Si}\left(sy_G - sy_{Si}\right)^2}{\sum_{i=1}^{n} H_{Si}}},$$

where the summations in the first term are over all strokes that have an inherent x scale, and the summations in the second term are over all strokes that have an inherent y scale.

5.3. Inconsistency of stroke placement within group

$$\varepsilon_{Gp} = \frac{1}{W_G + H_G}\sqrt{\frac{\sum_{i=1}^{m} L_{Si}\left[\left(dx_G - dx_{(G)Si}\right)^2 + \left(dy_G - dy_{(G)Si}\right)^2\right]}{L_G}}$$

where the summation is over all strokes in the group.

5.4. Deviations of stroke shape within group

$$\varepsilon_\delta = \frac{\sum_{i=1}^{n}\left(L_{Si}\cdot\frac{\delta_i}{L_{Si}}\right)}{L_G} = \frac{1}{L_G}\sum_{i=1}^{n}\delta_i,$$

where the summations are over all strokes in the stroke group. The first form shows that the simplified form (right) is equivalent to an archetype-path-length-weighted mean of maximum deviation in proportion to the archetype stroke path length.

5.5. Excess path length of strokes within group

$$\varepsilon_e = \frac{\sum_{i=1}^{n}\left(L_{Si}\cdot\frac{e_i}{L_{Si}}\right)}{L_G} = \frac{1}{L_G}\sum_{i=1}^{n}e_i$$

where the summations are over all strokes in the stroke group. The first form shows that the simplified form (right) is equivalent to an archetype-path-length-weighted mean of excess path length in proportion to the archetype stroke path length.

5.6. Errors of stroke continuity and connectivity

ε_{Gb} = *the number of broken stroke intersections*

ε_{Gc} = *the number of stroke clashes (unexpected contacts between strokes)*

ε_d = *the number of dropouts (undrawn vectors within strokes)*

6. Calculating proficiencies for vector proteins

6.1. The proficiency with which a vector protein performs the function of an archetype is calculated according to:

$$\Pi_P = \mathrm{Min}\{\Pi_G\}\cdot\left[\frac{1}{2}\right]^{\left\{\frac{\varepsilon_{Ps}}{\tilde{\varepsilon}_{Ps}}+\frac{\varepsilon_{Pp}}{\tilde{\varepsilon}_{Pp}}+\frac{\varepsilon_m}{\tilde{\varepsilon}_m}+\frac{\varepsilon_{Pb}}{\tilde{\varepsilon}_{Pb}}+\frac{\varepsilon_{Pc}}{\tilde{\varepsilon}_{Pc}}\right\}}$$

where each ε_i is dimensionless error measure (defined below), and $\tilde{\varepsilon}_i$ is a constant with a value that reflects the relative influence of ε_i on legibility. The minimum factor is the lowest proficiency of all stroke groups within the character.

The dimensionless error measures are defined as follows:

6.2. Inconsistency of group scaling within vector protein

$$\varepsilon_{Ps} = \frac{1}{sx_P}\sqrt{\frac{\sum_{i=1}^{m} W_{Gi}\left(sx_P - sx_{Gi}\right)^2}{\sum_{i=1}^{m} W_{Gi}}} + \frac{1}{sy_P}\sqrt{\frac{\sum_{i=1}^{n} H_{Gi}\left(sy_P - sy_{Gi}\right)^2}{\sum_{i=1}^{n} H_{Gi}}}$$

where the summations in the first term are over all stroke groups that have inherent x scale, and the summations in the second term are over all stroke groups that have inherent y scale.

6.3. Inconsistency of group placement within vector protein

$$\varepsilon_{Pp} = \frac{1}{W_P + H_P}\sqrt{\frac{\sum_{i=1}^{m} L_{Gi}\left[\left(dx_P - dx_{(P)Gi}\right)^2 + \left(dy_P - dy_{(P)Gi}\right)^2\right]}{L_P}}$$

6.4. Extraneous marks within vector protein

$$\varepsilon_m = \frac{1}{\mathrm{Min}\left\{L_S\right\}}\sum_{i=1}^{k}\left[\sum_{j=1}^{n_i-1}\sqrt{sx_P^2(x_{j+1}-x_j)^2 + sy_P^2(y_{j+1}-y_j)^2}\right]$$

where Min$\{L_S\}$ is the path length of the smallest stroke in the archetype. The outer summation is over all marks (coherent segments outside of strokes), and the inner summation is over vector endpoints (untransformed) in the ith mark (totaling n_i).

6.5. Errors of group connectivity

ε_{Pb} = *the number of broken group intersections*

ε_{Pc} = *the number of group clashes (unexpected contacts between groups)*

7. $\tilde{\varepsilon}$ values

Based on tests of legibility performed on vector proteins with controlled errors, *Stylus* uses the following $\tilde{\varepsilon}$ values:

$\tilde{\varepsilon}_{Gs} = 0.36$	$\tilde{\varepsilon}_{Gp} = 0.06$	$\tilde{\varepsilon}_{\delta} = 0.06$	$\tilde{\varepsilon}_{e} = 0.19$
$\tilde{\varepsilon}_{Gb} = 0.63$	$\tilde{\varepsilon}_{Gc} = 0.63$	$\tilde{\varepsilon}_{d} = 1.3$	
$\tilde{\varepsilon}_{Ps} = 0.63$	$\tilde{\varepsilon}_{Pp} = 0.13$	$\tilde{\varepsilon}_{m} = 3.6$	$\tilde{\varepsilon}_{Pb} = 0.63$
$\tilde{\varepsilon}_{Pc} = 3.1$			

8. Scoring of vector genomes/proteomes

8.1. The fitness of a genome/proteome is defined as:

$$f_{\text{genome}} = \frac{\text{Min}\left\{\Pi_{P,\phi=1}\right\} \cdot \left(1 + \sum_{\phi_i<1} \phi_i \cdot \text{Min}\left\{\Pi_{P,\phi_i}\right\}\right)}{c_{\text{fixed}} + c_{\text{base}} \cdot \text{bases} + c_{\text{unit}} \cdot (\text{total vector length})}$$

where c_{fixed} is the fixed 'overhead' cost, c_{base} is the cost of a DNA base, and c_{unit} is the per-unit-length cost of vectors (i.e, the cost of a small vector, medium and large vectors being proportionately more costly), *bases* is the length of the genome, *total vector length* is the entire path length of all genes, and ϕ_i is the likelihood (presumed constant under fixed global conditions) of a cell experiencing conditions where capability i (typically requiring multiple genes) is essential to survival. The first minimum is the lowest proficiency of the genes/proteins that contribute to *core* capabilities (where $\phi = 1$). The second is the lowest proficiency of the genes/proteins that contribute to the ith supplementary capability.

8.2. The current single-gene version of *Stylus* assumes that the gene under analysis contributes to core cell capabilities and limits these capabilities (by having the lowest value of Π_P). Since non-core capabilities are not under consideration in this context, *Stylus* calculates the fitness according to:

$$f_{\text{genome}} = \frac{\Pi_P}{c_{\text{fixed}} + c_{\text{base}} \cdot \text{bases} + c_{\text{unit}} \cdot (\text{total vector length})}$$

where *bases* means the full length of the open reading frame, including start and stop codons, and *total vector length* means the combined length of all vectors in the vector protein.

Since the absolute value of costs (and therefore of fitness) is arbitrary, costs are scaled such that a hypothetical reference gene has unit fitness. The reference gene is taken to have the following properties: 1000 bases; 500 vector units; $\Pi_P = 0.50$.

Based on these values, and on an assumed 20-fold higher total cost of vectors relative to bases (corresponding to a 20-fold higher cost of protein relative to DNA[1]), we have: $c_{\text{base}} = 2.38 \times 10^{-5}$ and $c_{\text{unit}} = 9.52 \times 10^{-4}$.

---

[1] Neijssel OM, Joost Teixeira de Mattos M, Tempest DW (1996) Growth yield and energy distribution. In: *Escherichia coli* and *Salmonella*– Cellular and Molecular Biology (2nd ed.) Neidhardt FC, editor in chief. ASM Press, Washington, D.C.
